# Bone Marrow Microenvironment-On-Chip for Culture of Functional Hematopoietic Stem Cells

**DOI:** 10.3389/fbioe.2022.855777

**Published:** 2022-06-20

**Authors:** Azmeer Sharipol, Maggie L. Lesch, Celia A. Soto, Benjamin J. Frisch

**Affiliations:** ^1^ Department of Biomedical Engineering, University of Rochester, Rochester, NY, United States; ^2^ Center for Musculoskeletal Research, University of Rochester Medical Center, Rochester, NY, United States; ^3^ Department of Microbiology and Immunology, University of Rochester Medical Center, Rochester, NY, United States; ^4^ Department of Pathology and Laboratory Medicine, University of Rochester Medical Center, Rochester, NY, United States; ^5^ Wilmot Cancer Institute, University of Rochester Medical Center, Rochester, NY, United States

**Keywords:** bone marrow, hematopoietic stem cell, bone marrow microenvironment, bone marrow on a chip, tissue engineering, 3D chip, osteoblast, mesenchymal stem cell

## Abstract

Hematopoiesis takes place in the bone marrow and is supported by a complex cellular and molecular network in the bone marrow microenvironment. Commonly used models of the human bone marrow microenvironment include murine models and two-dimensional and three-dimensional tissue cultures. While these model systems have led to critical advances in the field, they fail to recapitulate many aspects of the human bone marrow. This has limited our understanding of human bone marrow pathophysiology and has led to deficiencies in therapy for many bone marrow pathologies such as bone marrow failure syndromes and leukemias. Therefore, we have developed a modular murine bone marrow microenvironment-on-chip using a commercially available microfluidic platform. This model includes a vascular channel separated from the bone marrow channel by a semi-porous membrane and incorporates critical components of the bone marrow microenvironment, including osteoblasts, endothelial cells, mesenchymal stem cells, and hematopoietic stem and progenitor cells. This system is capable of maintaining functional hematopoietic stem cells *in vitro* for at least 14 days at frequencies similar to what is found in the primary bone marrow. The modular nature of this system and its accessibility will allow for acceleration of our understanding of the bone marrow.

## Introduction

The adult hematopoietic system arises from the hematopoietic stem cell (HSC) that resides in and is supported by the HSC niche within the bone marrow. The HSC niche is a complex, interconnected network of cellular and molecular components within the bone marrow microenvironment (BMME) (Frisch, 2019). A major challenge that hampers progress in the field of the BMME is the lack of a reliable model system that can recapitulate the human bone marrow. Murine models have been used extensively because they allow for *in vivo* studies and powerful genetic tools. However, mouse models often fail to recapitulate the human environment. Conventional 2D cultures are another common approach used to investigate the human BMME for the study of normal hematopoiesis and hematopoietic malignancies (Congrains et al., 2021). However, 2D cultures lack the ability to completely elucidate bone marrow pathophysiology and are typically limited to 2–3 different cell types. Overall, the inadequacies of conventional preclinical models contribute to a relatively poor understanding of the human BMME and the large attrition rates of Phase II and III of clinical trials (Arrowsmith and Miller, 2013).

In recent years, *in vitro* 3D models of various organ systems have been developed to facilitate basic biology research and drug development and screening. The development of these preclinical disease models is beneficial in understanding complex microenvironmental interactions with the potential implications of discovering novel treatment strategies (Duarte et al., 2018). The three-dimensional culture strategy is advantageous as it can provide an avenue to recapitulate the complex tissue microstructures while reducing the exhaustive requirements of animal models (Huh et al., 2011; Low and Tagle, 2017a; Low and Tagle, 2017b; Osaki et al., 2018; Kelm et al., 2019; Low et al., 2020). Therefore, a 3D microphysiological system will advance the field of BMME research by providing a viable platform for applications in mechanistic studies and drug discovery.

Several groups have shown the use of fibrin-based hydrogels in microfluidics platforms and the incorporation of a dynamic vasculature component were able to sustain HSCs *in vitro* (Chou et al., 2020; Nelson et al., 2021). Synthetically derived scaffolds such as PEG crosslinked with MMP-degradable peptide or tethered with adhesive ligands show promising evidence of HSC sensitivity towards culture dimension and flow conditions (Bray et al., 2017; Rödling et al., 2017). Although these developments provide valuable insights into establishing niche incorporated models, the outcomes of these models are limited to studying myelotoxicity towards drugs and radiation rather than insights into the mechanisms of HSC/niche interactions. Most of the current models lack major BMME components such as osteoblastic cells. Osteoblasts are important in establishing the endosteal HSC niche compartment, regulating bone remodeling and promoting the survival of lymphoid progenitors (Duarte et al., 2018). Our group and others have shown that leukemic cell interactions with the BMME, specifically through osteoblastic crosstalk, can drive leukemic progression and development (Frisch et al., 2012; Staversky et al., 2018; Ackun-Farmmer et al., 2021). Therefore, it is crucial to incorporate the osteoblastic components in addition to the vascular niche to generate a reliable 3D model of the BMME.

In this study, we generated a 3D model of the murine BMME with functional osteoblastic, endothelial, and stromal cell compartments to recapitulate the endosteal niche that is favorable for HSC survival. We used a commercially available microfluidics system, the Human Emulation System from Emulate Bio, and fibrin hydrogel scaffolding to culture HSC and performed competitive transplant assays to evaluate the self-renewal property of BMME-on-chip grown HSCs. The development of this preclinical model is valuable for the study of BMME pathologies, BMME-hematopoietic cells crosstalk, and identifying novel therapeutic targets.

## Murine BMME-On-Chip

**FIGURE 1:**
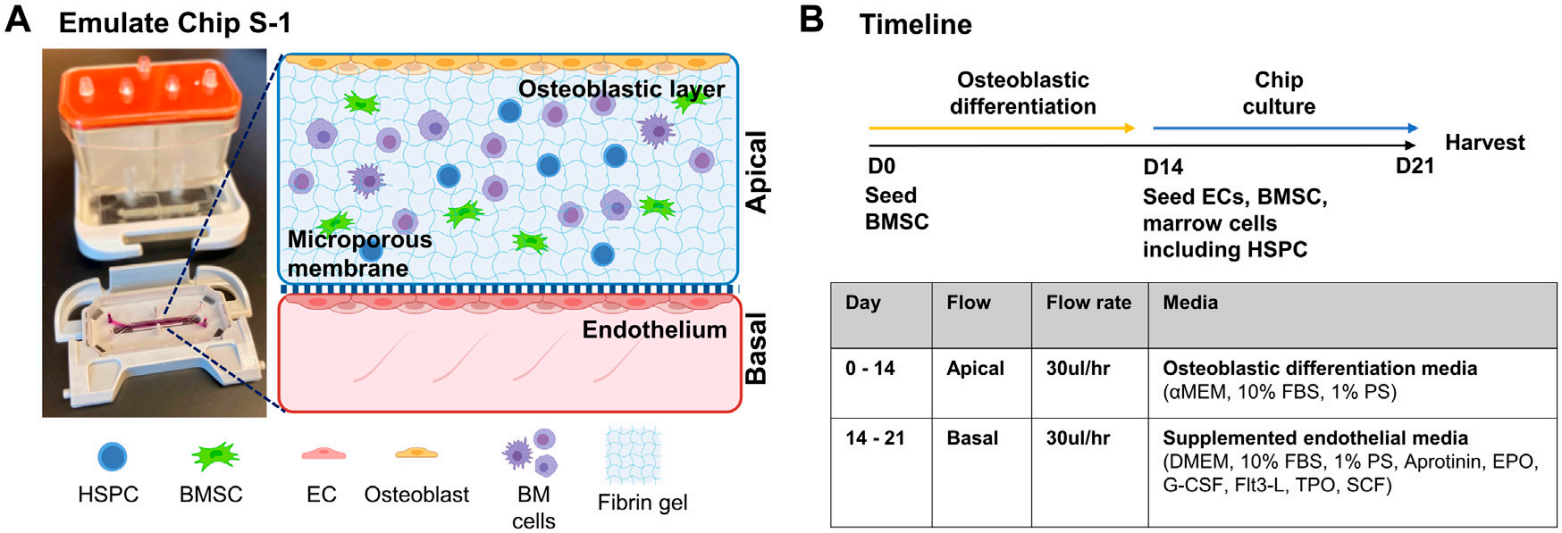
BMME-on-chip with osteoblastic, endothelium layer and whole bone marrow cells. **(A)** Diagram of BMME-on-chip and **(B)** culture timeline. Illustrated using Biorender.com.

Our goal was to generate a BMME-on-chip with niche components that are reliable, cost-effective, and easily adopted across research laboratories (Figure 1). The development of in-house microfluidics models is beneficial in generating complex architecture for *in vitro* systems; however, it can be time-consuming and limited to a specific laboratory. In this study, we utilized Emulate Chip S-1™ to provide the geometry and mechanical support to culture cells. The chip contains two channels separated by a semi-porous membrane which allows for the physical separation of cellular compartments. The chip is assembled with a housing system, Pod™, that contains input and output reservoirs for both channels and is connected to a module that automatically regulates fluid flow in each channel. The validated setup allows for efficient culturing of cells without the need for frequent media changes, troubleshooting flow modules, or optimizing microfluidics architecture.

**FIGURE 2:**
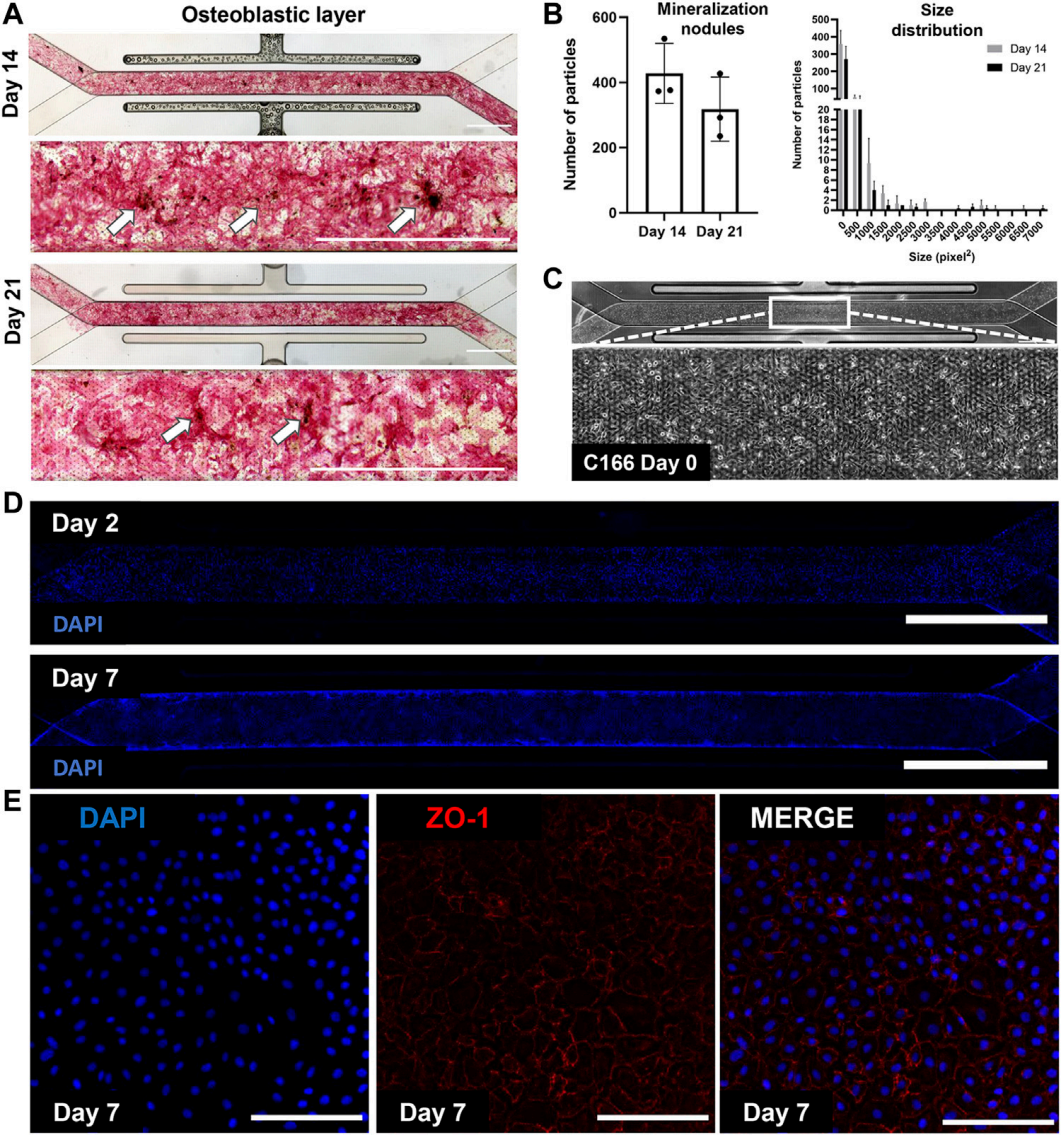
Mineralized osteoblastic layer and C166 endothelium formation with tight junction expression. **(A)** Representative images of positive mineralization by alkaline phosphatase and Von Koss staining on D14 and D21. Arrows indicate calcium nodules and scale bar = 2 mm. **(B)** Quantification of mineralization using particle analysis *via* ImageJ of D14 and D21 mineralized chips. **(C)** Representative image of C166 monolayer on the chip on D0 post-seeding. **(D)** Representative fluorescent images of C166 cells (nuclei, DAPI) on Day 2 and Day 7. Scale bar = 2 mm. **(E)** C166 endothelium formed cobblestone morphology and expressed tight junction protein, ZO-1 (red). Scale bar = 100 μm).

We have cultured bone marrow stromal cells (BMSC) isolated from wild type C57BL/6 J mice into the apical channel of Chip S-1 to create a supportive osteoblastic layer (Figure 2)**.** Upon 14 days of culture under a continuous flow rate of 30 μL/h with osteoblast differentiation media, robust differentiation and mineralization were observed in chips, as shown by positive alkaline phosphatase and Von Kossa staining and quantification (Figure 2B). On D14, we introduced mouse endothelial C166 cells on the basal surface of the membrane to generate a supportive vascular endothelial monolayer (Figure 1C). C166 cells completely occupied the membrane of the channel at D2 and surrounded the walls of the channel by D7, as indicated by positive nuclei staining (DAPI, Figure 2D). On D7, we found robust endothelium formation as shown by cobblestone morphology and tight junction protein expression, zonula occludens-1 (ZO-1) (Figure 2E). On D14, a cell-laden fibrin hydrogel was introduced in the apical compartment containing BMSC and whole bone marrow cells. The chip was then subjected to a culture regime of supplemented endothelial media in the basal channel at 30 ul/hr. The chip was grown for 7 days before harvesting on D21 for analysis (Figure 1B).

**FIGURE 3:**
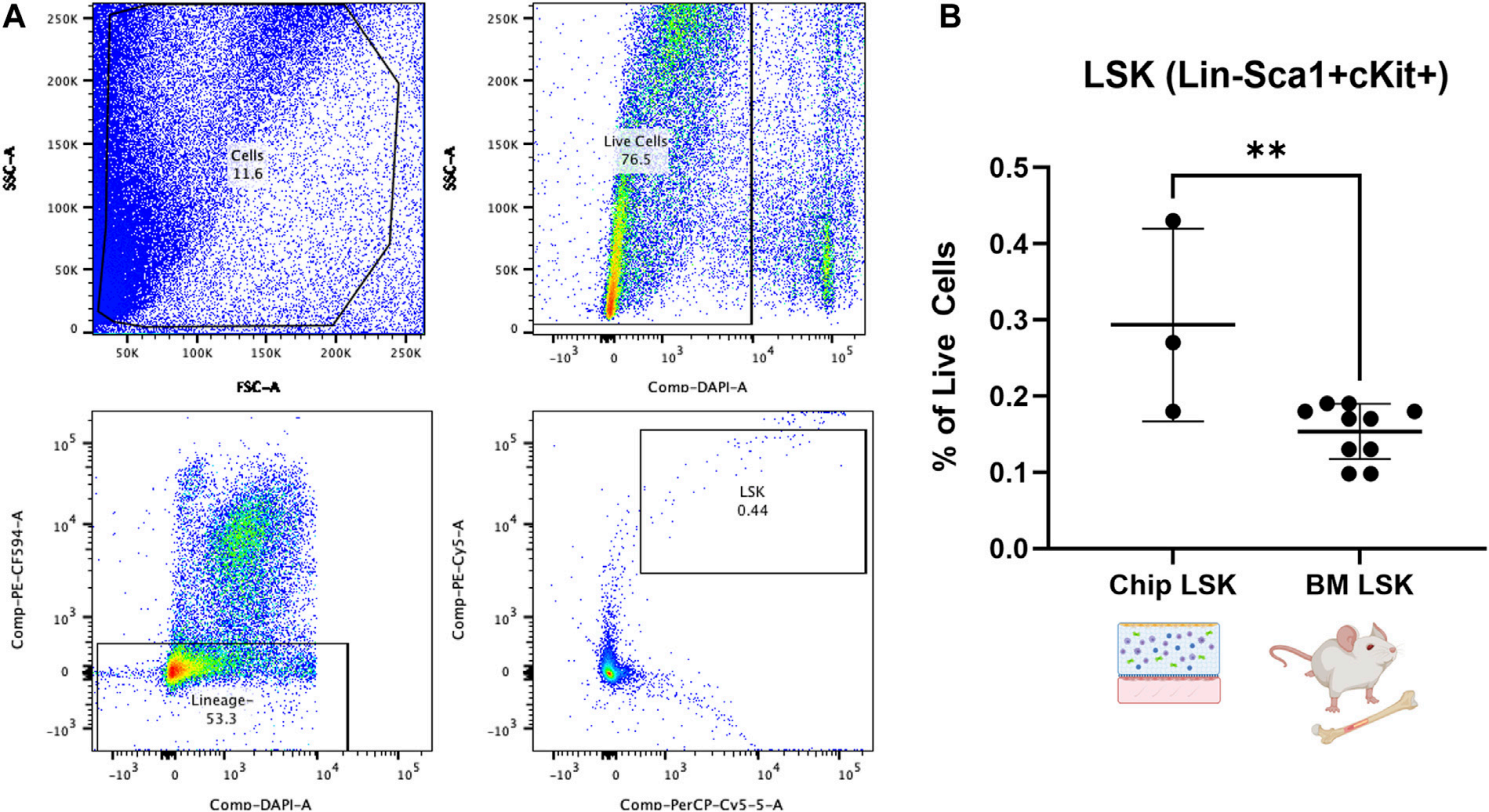
Flow cytometric analysis of HSPC cells (LSK: Lineage-, Sca1+, cKit+) from whole bone marrow cells in BMME-on-Chip. **(A)** Gating strategy for analysis of LSK cells using FlowJo v10. **(B)** Percent of LSK in live cells population in the chip on D21 and control C57Bl/6J BM at 6–8 weeks of age (Mean, SD). Statistical analysis was performed using an unpaired *t*-test. **: *p* ≤ 0.01. BM: Bone marrow.

We expect that the BMME-on-chip can provide a conducive environment for marrow cell culture due to the multifaceted culture system. In particular, we are interested in investigating the fate of hematopoietic stem cell and progenitor cells (HSPC) within the marrow compartment after 7 days of chip culture. Therefore, on D21, we harvested cells from BMME-on-chip and control mice BM and stained them for lineage (Gr1, CD3e, B220, and Ter119) and HSPC (Sca1 and cKit) markers^7^ (Figure 3A). HSPC (LSK, Lin−Sca+cKit+) population was preserved in healthy BMME-on-chip after 7 days of culture at ∼0.3% of total cells. This is two-fold greater than levels to *in vivo* of ∼0.15% (Figure 3B). The data strongly suggest that BMME-on-chip is a reliable surrogate for HSPC maintenance *in vitro.*

**FIGURE 4:**
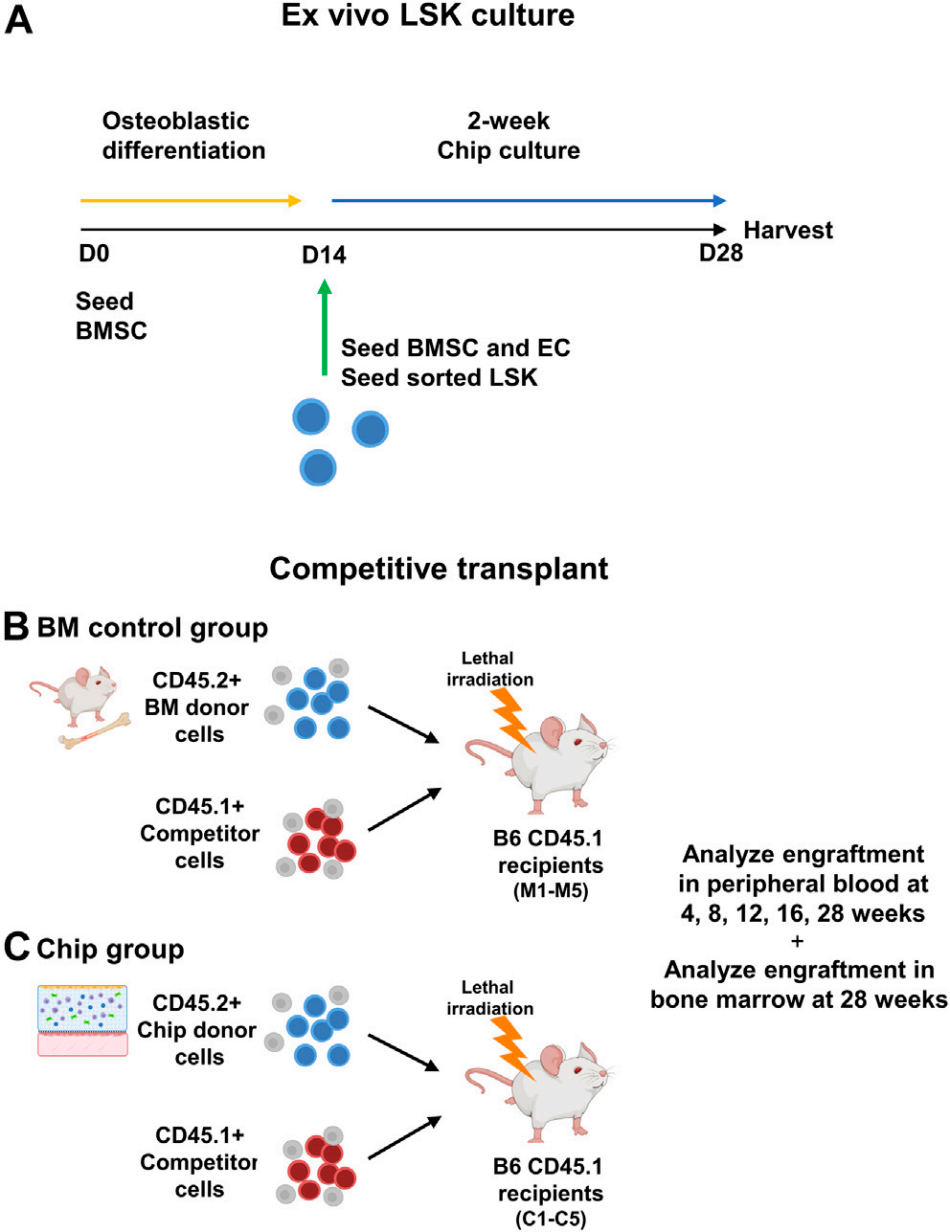
Diagram of *ex vivo* culture of sorted LSK cells in BMME-on-chip and competitive transplant. **(A)** CD45.2+ LSK cells were sorted using flow cytometry and cultured with BMSC in hydrogel for 14 days in BMME-on-chip. **(B)** Functional analysis using competitive transplant assay of bone marrow and chip grown LSK CD45.2+ donor into recipient B6 CD45.1 **(C)** with CD45.1+ competitor cells. Illustrated using Biorender.com.

**FIGURE 5:**
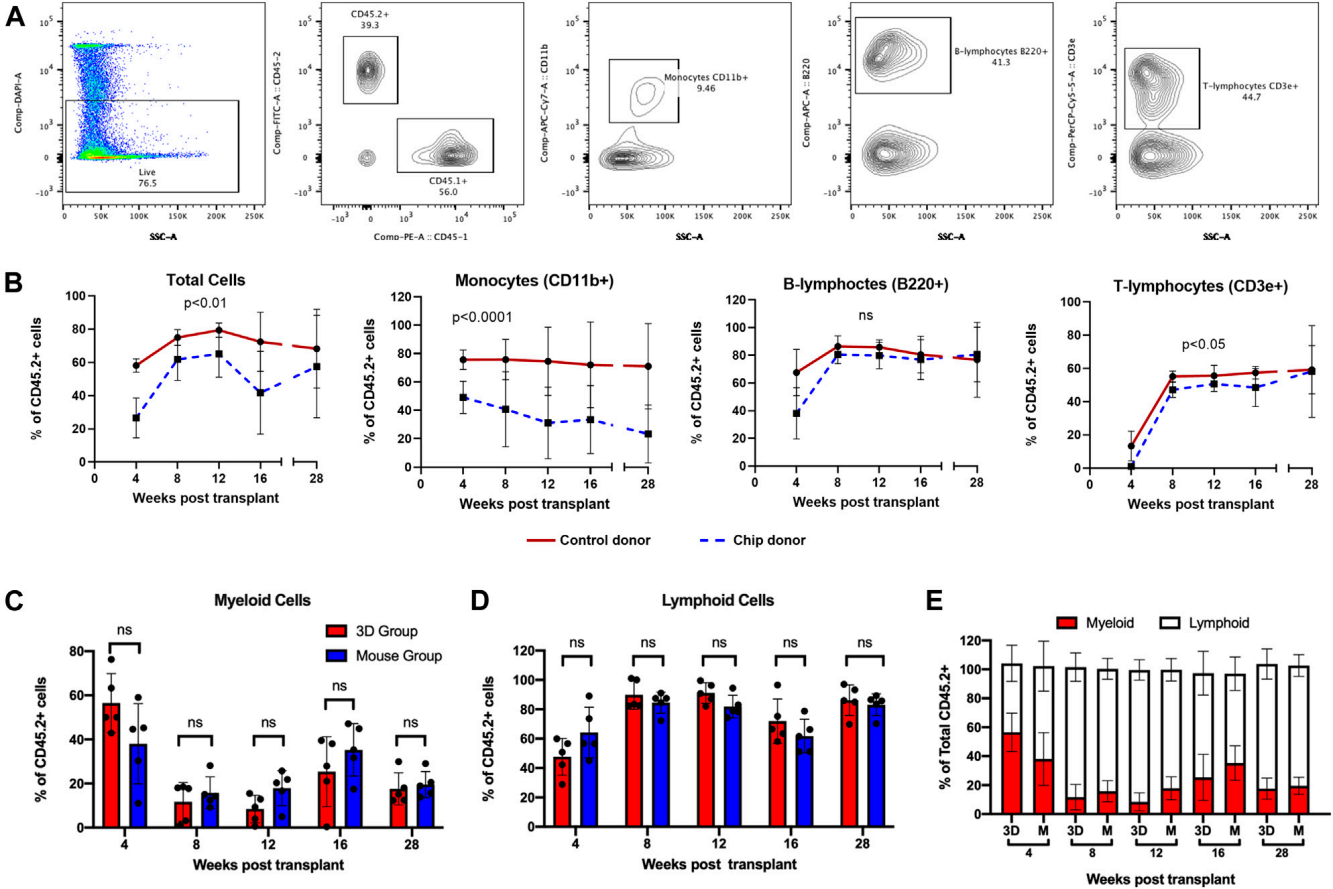
Differentiation of LSK transplanted into mature peripheral blood phenotypes over time. **(A)** Gating strategy for flow cytometry analysis at 4, 8, 12, 16, and 27-weeks post-transplantation. CD11b+, B220+, and CD3e+ were gated from CD45.2+ live cells. **(B)** Quantification of CD45.2+ donor cells in total cell, CD11b+ monocyte, B220+ B-lymphocyte, and CD3e+ T-lymphocyte populations. **(C)** Chip grown CD45.2+ cells gave rise to myeloid cells at a similar frequency to mouse control. Quantification was performed by gating CD11b+ cells within CD45.2+ populations. **(D)** Chip CD45.2+ cells engrafted into mature lymphoid populations similar to the mouse control group. Data are shown as combined percentage of B220+ and CD3e+ cells in CD45.2+ cells. The gating strategy for **(C** and **D)** is provided in Supplementary Figure S4. **(E)** Graph showing the percentage of myeloid and lymphoid cells in total CD45.2+ peripheral blood cells. All flow analysis was performed using FlowJo v10. Statistical analysis for **(B)** was performed using restricted maximum likelihood (REML). N = 5 per group. Statistical analysis for **(C,D)** was performed using multiple Mann–Whitney tests.

HSPC are self-renewing and multipotent cells able to reconstitute the BM and differentiate into mature blood cells. Therefore, the standard functional analysis of HSPC is *via* a mouse BM transplantation model, whereby experimental HSPC are transplanted into myeloablated mice with the addition of competitor cells as an internal control (Figure 4). Sorted LSK cells from C57BL/6 J mice (CD45.2+) were grown in chips with CD45.2+ BMSCs for 14 days (Figure 4A). The whole content of each chip was mixed with whole BM cells from CD45.1+ PepBoy B6 mouse as a competitor and transplanted into a lethally irradiated CD45.1+ PepBoy B6 mice (Figures 4B–C). Equal numbers of CD45.2+ freshly isolated bone marrow cells were also mixed with CD45.1+ BM cells and transplanted into lethally irradiated CD45.1+ recipients as controls. We analyzed peripheral blood (PB) cells *via* flow cytometry at multiple timepoints after the transplant. We found that CD45.2+ chip LSK were able to successfully engraft in recipient mice at similar levels compared to control HSPC from whole bone marrow (Figure 5). In particular, chip LSK gave rise to ∼40–60% of total PB cells, which include myeloid populations, B lymphocytes, and T lymphocytes (Figure 5B). To confirm multilineage differentiation capacity, we compared the frequency of myeloid and lymphoid cells that were derived from CD45.2+ cells in primary recipients. CD45.2+ cells harvested from the BMME-on-chip generated myeloid cells at similar frequencies compared to cells harvested from freshly isolated bone marrow (C). At 28-weeks post-transplant, myeloid frequencies were at ∼20% in peripheral blood of both chip and mouse recipients (D), while lymphoid populations were at ∼80% (D-E). Thus, our findings suggest that chip-derived cells have the equivalent potential for generating myeloid and lymphoid cells to the mouse control group.

**FIGURE 6:**
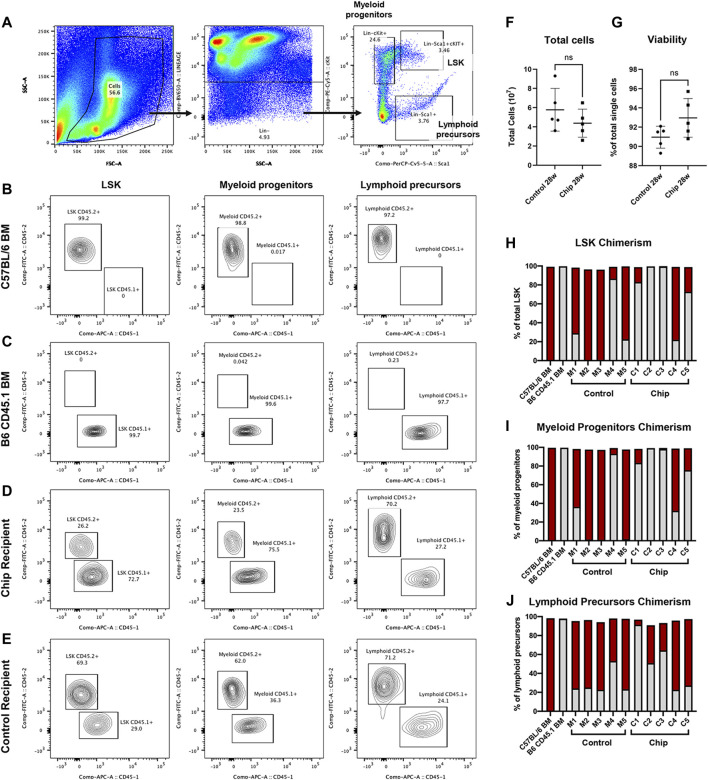
Engraftment and repopulation of HSPC, myeloid progenitor, and lymphoid precursor populations in bone marrow by BMME-on-chip grown LSK cells. **(A)** Bone marrow of recipient mice was isolated and analyzed using flow cytometry for lineage markers, stem cell markers Sca1+ and cKit+. **(B–E)** Representative contour plots of CD45.1+ and CD45.2+ in LSK (Lin-cKit + Sca1+), myeloid (lin-cKit+), and lymphoid (lin-Sca1+) cells in bone marrow of **(B)** CD45.2+ C5BL/6J, **(C)** B6 CD45.1+, **(D)** chip transplant recipient, and **(E)** mouse marrow control recipient. **(F)** Quantification of total bone marrow cells after isolation. **(G)** Viability isolated bone marrow cells using flow cytometry (negative DAPI staining). Statistical analysis for F and G was performed using an unpaired *t*-test. ns = no significance, error bar = SD. **(H–J)** Chimerism of LSK **(H)**, myeloid **(I)**, and lymphoid **(J)** cells indicating repopulation of CD45.2+ donor cells and competitor CD45.1+ cells in recipient mice. Data show the percent of total cells analyzed using flow cytometry of bone marrow from each recipient mouse. In particular, control recipients M1, M4, and M5, and chip recipients C1, C4, and C5 show chimerism in LSK populations.

Bone marrow engraftment was analyzed by detecting chimerism of CD45+ HSPC populations at a very late timepoint, 28-weeks post-transplant. Flow cytometry was used to quantify chimerism in LSK population and lineage-committed myeloid progenitors (Lin−cKit+) and lymphoid precursors (Lin−Sca1+) (Figures 6A–E). Chimerism was observed in all three populations in the majority of the mice transplanted with HSPC from bone marrow and chip (Figures 6H–J). In particular, CD45.2+ donor cells were observed at a high percentage in LSK populations of control recipients M1, M4, and M5 and in chip recipients C1, C4, and C5 (Figure 6H). All of the five control recipients showed engraftment of CD45.2+ donor cells, with M2 and M3 having a majority of the repopulated LSK derived from donor cells. On the other hand, two out of the five chip recipients showed low to no repopulation of LSK by donor cells. Myeloid and lymphoid committed cells also showed chimerism as indicated by CD45.2+ cells in control and chip recipients (Figures 6I and J).

**FIGURE 7:**
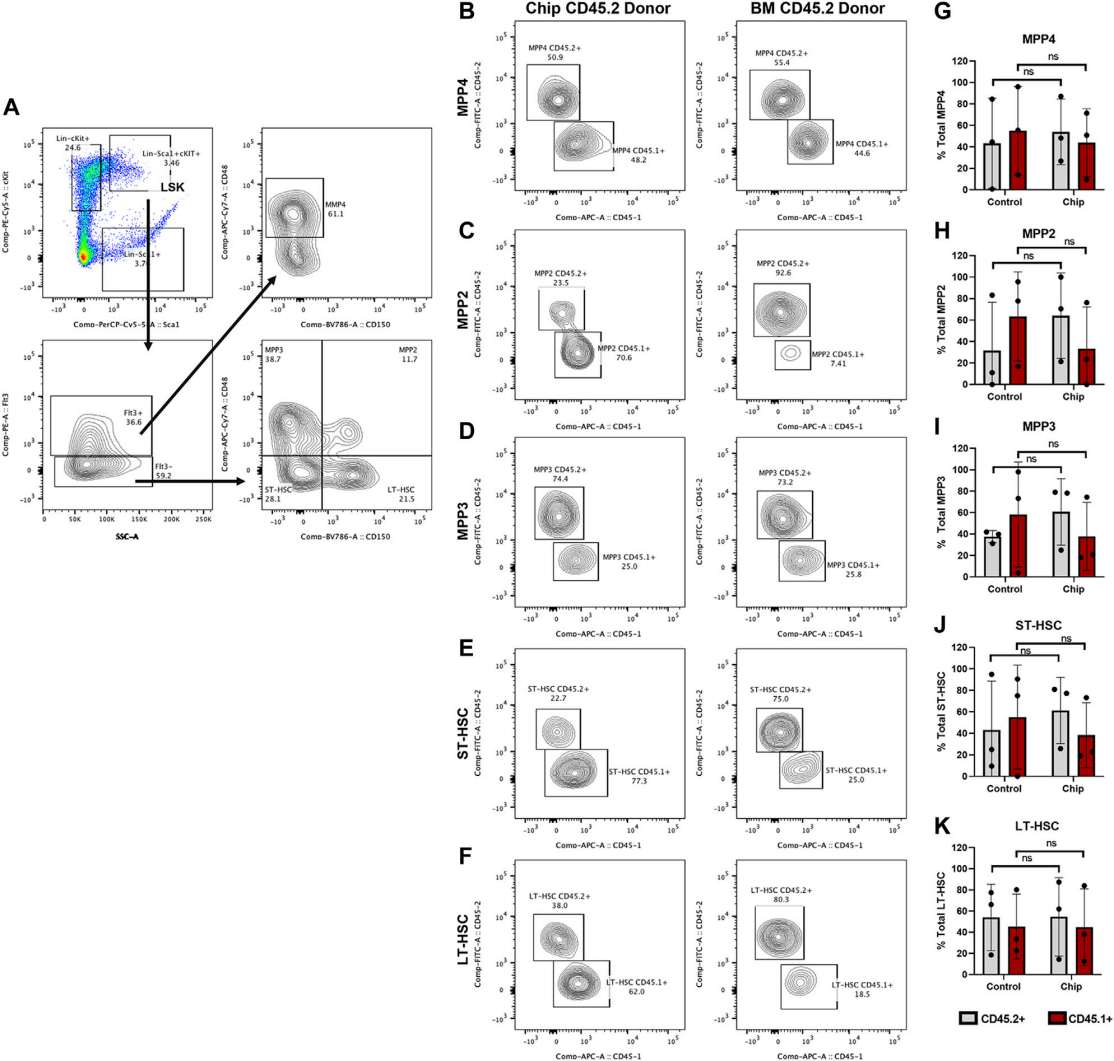
Repopulation of myeloid-biased MPP2+ and MPP3+, lymphoid-biased MPP4+ multipotent progenitor and HSC populations by BMME-on-chip donor cells. Subpopulations of specific progenitor cells and short-term and long-term repopulating HSC (ST-HSC and LT-HSC) within the LSK populations were analyzed for mice recipients with CD45.2+ and CD45.1+ chimerism (M1, M4, M5, C1, C4, and C5). **(A)** Flow cytometry gating of LSK subpopulations. **(B–K)** Representative contour plots and quantification of donor and competitor cells in LSK subpopulations. Statistical analysis was performed using multiple Whitney–Mann tests. *N* = 3 per group. Error bars = standard deviation, ns = non-significant.

HSPC subpopulations were further analyzed within the LSK cells in recipient mice with high chimerism (M1, M4, M5, C1, C4, and C5) (Figure 7). Our findings showed CD45.2+ repopulation of myeloid-biased (MPP2 and MPP3) and lymphoid-biased (MPP4) multipotent progenitor populations in chip recipients at similar levels to control recipients (Figures 7B–D and G–I). In addition, CD45.2+ short-term and long-term repopulating HSC (ST-HSC) and (LT-HSC) were also observed at high levels, 40% for both groups, in chip recipients. Overall, the majority of mice transplanted with HSPCs obtained from BMME-on-chip cultures maintained similar distribution of HSPC subpopulations even through this very long-term engraftment.

**FIGURE 8:**
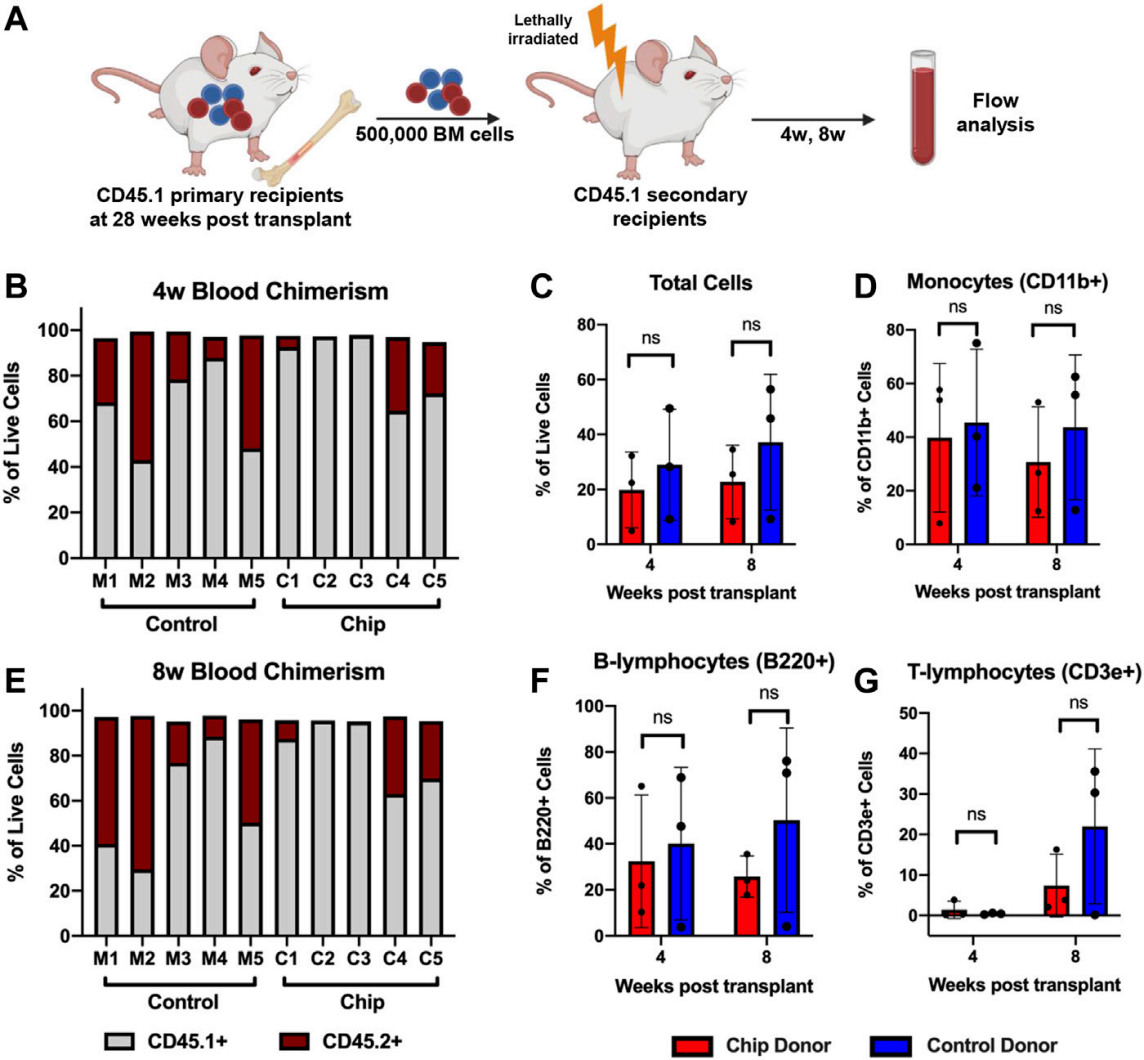
Repopulation of peripheral blood population in secondary transplant recipients shows maintenance of long-term HSPC function after BMME-on-chip culture. **(A)** Diagram of secondary transplant of lethally irradiated mice with bone marrow cells from primary recipients at 28 weeks post-transplant. Peripheral blood was analyzed using flow cytometry at 4w and 8w post-secondary transplant. **(B,C)** Flow cytometry data showing chimerism of CD45.2+ donor cells in total live cells in peripheral blood at 4w and 8w. Consistent with bone marrow engraftment data, C2 and C3 do not have CD45.2+ engraftment. **(D–F)** Flow analysis of total cells, monocytes, and lymphocytes from chip recipients with CD45.2+ engraftment (C1, C4, and C5) compared to mouse recipients (C1, C4, and C5). Flow analyses were performed using FlowJo V10. Statistical analysis for **(D–F)** was performed using an unpaired *t*-test. *N* = 3. ns = non-significant.

To investigate long-term HSPC function, a secondary transplant assay was performed by injecting bone marrow cells isolated from primary recipient mice into myeloablated secondary recipients (Figure 8A). PB was analyzed at 4- and 8-weeks post-transplant. Consistent with engraftment data in the bone marrow, only C1, C4, and C5 samples from the chip group showed engraftment of CD45.2+ cells at 4- and 8-weeks (Figures 8B and C). Overall, CD45.2+ chip-derived cells comprised approximately 20% of total live cells at 4- and 8-weeks post-transplant (Figure 8D). Although the mouse group showed higher trends of engraftment, the comparison showed no statistical significance between the groups. Importantly, long-term differentiation of HSPC into mature blood populations was maintained as indicated by monocyte and lymphocyte populations that were CD45.2+ post-transplant (Figures 8E and F).

Our findings currently showed that chip grown HSPC are self-renewing and capable of regenerating the hematopoietic system, which current 3D systems have not been able to demonstrate. This reliability of the chip to provide a permissive microenvironment for HSC maintenance makes it a valuable platform for future work in studying HSPC interactions with the BMME and BM pathologies. Here, we present step by step methodologies to generate BMME-on-chip, including protocols for Emulate Chip preparation, cell culture, MSC isolation, chip characterization, and competitive transplant assay.

## Materials

### Reagents

 αMEM (1x) (Gibco, Cat #A10490-01).

DMEM (1x) (Corning, Cat #10-013-CV).

FBS (Gibco, Cat #26140-079).

PBS 1X (Corning, Cat# 21-040-CV).

0.25% Trypsin-EDTA (1x) (Gibco, Cat #25200-056).

Pen–Strep (Penicillin–Streptomycin, Sigma-Aldrich, Cat #P4).

L-ascorbic acid (Krackeler, 45-A4544).

Glycerol 2-phosphate disodium salt hydrate (Krackeler,    G9422).

Recombinant mouse EPO (Fisher, 587602).

Recombinant murine G-CSF, murine (PeproTech, Cat# 50-   813-314.

Recombinant murine FLT3-ligand (PeproTech, Cat#    250-31L).

Recombinant murine TPO (PeproTech, Cat# 50-813-680).

Recombinant murine SCF (PeproTech, Cat#250-03).

Recombinant murine IL3 (PeproTech, Cat# 213-13).

Recombinant murine IL6 (PeproTech, Cat# 216-16).

Fibrinogen, lyophilized (Sigma, Cat# 341578).

Thrombin, lyophilized (Sigma, Cat# 605195).

Aprotinin, lyophilized (Sigma, Cat# A3426).

Matrigel^®^ (Corning, Cat# 354234) - for Chip S-1 activation.

Collagen type I solution, 8–11 mg/mL (Corning, Cat# 354249)    - for Chip S-1 activation.

Nattokinase (Pure Formulas, Cat# PE 1801).

Collagenase type 1 (Gibco, Cat#17-018–029).

HEPES (Fisher BioReagents, BP299100).

10% Neutral buffered formalin (VWR, Cat# 89370-094).

Silver nitrate solution, AgNO_3_, 99.9+% (AlfaAesar,    Cat#11414).

Fast Red Violet LB salt (Sigma, Cat# F3381).

Tris-HCl at 0.2M, pH 8.3 (Roche, Cat# 10812846001).

N,N-Dimethylformamide (DMF) (AlfaAesar, Cat#A13547).

Naphthol AS-MX phosphate, (Sigma, Cat# N4875).

UltraComp Beads (Invitrogen, Cat# 501129040).

Distilled water.

Ammonium chloride (NH4Cl) (Krackeler, Cat# 45-213330).

Ethylenediaminetetraacetic acid (EDTA) (Sigma, Cat# E4884).

Sodium bicarbonate (NaHCO3) (Sigma, Cat#S6014).

### Plastics

   500 mL Vacuum filtration flask 0.22 μm (LPS CellPro, Cat#    V50022).

250 mL Vacuum filtration flask 0.22 μm (LPS CellPro, Cat#    V25022).

50 mL Sterile-HV sterile centrifuge tube PVDF membrane,    0.45 μm, top filter unit (Milipore, Cat# SE1M003M00).

Eppendorf tubes 1.5 mL (FisherSci, 02-682-002).

40 μm cell strainer (Fisher, 08-771-1).

6-well cell culture plate (Nest, Cat# 703001).

Cell culture dish, 150 mm × 25 mm (Nest, Cat# 715001).

17 × 75 mm culture tubes with closures (VWR, 60818-496).

### Mice

 All mice and protocols used in the experiments were approved by the Institutional Animal Care and Use Committee, the University Committee on Animal Resources at the University of Rochester School of Medicine Dentistry (URMC). Animals were housed in the Vivarium facility at URMC.

C57BL/6 J mice, males, 8–12 weeks of age (Jackson Laboratories, Strain# 000664).

B6 CD45.1+ mice, males, 8–12 weeks of age (B6.SJL-*Ptprc* ^*a*^ *Pepc* ^*c*^/BoyJ) (Jackson Laboratories, Strain# 002014).

### Cells

 C166 cell line (ATCC Cat# CRL-2581).

### Emulate Human Emulation System

 Chip S-1™

Pod™ Portable Module.

Zoë™ Culture Module.

Orb™ Hub Module.

### Equipment

 Cell culture incubator set to 37°C. 5% CO_2_, 95% humidity.

Water bath set to 37°C.

Cytation 5 Imaging System.

^137^Cs radiation source (GAMMACELL- 40).

### Reagent Setup

#### FBS Heat Inactivation

1) Thaw frozen FBS overnight at 4°C.
2) Once thawed, bring serum to room temperature.
3) Place serum in a 56°C water bath for 30 min and swirl the bottle every 5 min.
4) Make aliquots of heat-inactivated serum and store at −20°C until use.

#### Complete media (10% FBS + 1% PS)

1) Thaw 5 mL Pen–Strep in a 37°C water bath.
2) For 500 mL of complete media, combine 5 mL Pen–Strep, 50 mL of heat-inactivated FBS, and 445 mL of αMEM.
3) Vacuum filter complete media using a 500 mL vacuum filtration flask.
4) Make 50 mL aliquots of media in conical tubes.
5) Warm up to 37°C prior to use or store in a fridge for short term use or in a freezer for long term use.

#### Osteoblast Differentiation Media

1) Make 1M stock solution of glycerol 2-phosphate disodium salt hydrate in sterile water. Store stock in smaller aliquots in a −20°C freezer.
2) For 200 mL of differentiation media, combine 10 mg of L-ascorbic acid, 2 mL of 1M thawed glycerol solution, and 200 mL of complete media.
3) Filter media using a 250 mL vacuum filtration system. Warm media to 37°C before use or store in a freezer or fridge. The media will have a final concentration of 50 μg/mL of L-ascorbic acid and 10 mM of glycerol 2-phosphate disodium salt hydrate.
3) Gas equilibrate with 0.45 μm Steriflip-HV and warm up media for at least 1 h at 37°C prior to use with chips.

#### Endothelial Media (10% FBS + 1% PS) for C166 Culture

1) For 500 mL of complete endothelial media, combine thawed 5 mL Pen–Strep, 50 mL of heat-inactivated FBS, and 445 mL of DMEM.
2) Warm up to 37°C prior to use or store at 4°C for short term use.

#### Supplemented DMEM Media

1) For 50 mL of media combine complete endothelial media (10% FBS +1% PS) with the following concentration of cytokines: EPO 20 ng/mL, G-CSF 1 ng/mL, FLT3L 100 ng/mL, TPO 100 ng/mL, SCF 50 ng/mL, IL-3 10 ng/mL, and IL-6 10 ng/mL.
2) Warm up to 37°C prior to use or store in a fridge for short term use or in a freezer for long term use.
3) Gas equilibrate with 0.45 μm Steriflip-HV and warm up media for at least 1 h at 37°C prior to use with chips.

#### Fibrin Gel Components

1) Fibrinogen: resuspend at 25 mg/mL in sterile water and make aliquots of 100 μL or 200 μL. Store in a −20°C freezer.
2) Aprotinin: resuspend at 1 mg/mL in sterile water and make aliquots of 12.5 μL or 25 μL. Store in a −20°C freezer.
3) Thrombin: resuspend at 1 U/μL in sterile water and make aliquots of 1 μL. Store in a −20°C freezer.
4) Collagen Type I solution: make aliquots of 10–25 μL. Store in a fridge at 4°C.

#### Chip Digestion Solution

1) Warm up complete DMEM in a 37°C water bath for at least an hour.
2) Prepare 5 mL of digestion solution with final concentrations of 1 mg/mL nattokinase, 25 mM HEPES, and 1 mg/mL collagenase type 1.

#### Flow Cytometry Staining Buffer (2% FACS Buffer)

1) Prepare 2% FACS buffer by combining thawed 10 mL of heat-inactivated FBS with 500 μL of 1X PBS.
2) Store in a fridge at 4°C. Use cold buffer for staining and bone marrow dissociation.

#### Alkaline Phosphatase and VonKossa Staining

1) Prepare fresh alkaline phosphatase staining substrate by dissolving 0.005 g of Napthol AS MX with 200 μL of DMF in an Eppendorf tube. Combine 25 mL of Tris HCL pH 8.3, 0.2 M with 25 mL of distilled water. Combine all reagents together with 0.03 g of red violet LB salt.
2) Prepare 2.5% silver nitrate Von Kossa staining solution by dissolving 2.5 g of silver nitrate with 100 mL of distilled water.

#### Red Blood Cell Lysis Buffer

1) Prepare a solution of 156 mmol/L NH4Cl, 127 μmol/L EDTA, and 12 mmol/L NaHCO3.

## Methods and Protocols

### BMME-On-Chip Preparation

#### Day-14: (I) Primary Bone Marrow Cell Isolation

1) Obtain marrow cells from long bones of the hind limbs of mice and flush out marrow using a 25-gauge needle and FACS buffer.
2) Dissociate marrow with a 16-gauge needle and filter with a 40 μm cell strainer. Collect in a 50 mL conical tube.
3) Fill the conical tube to 25 mL with FACS buffer and centrifuge the cell solution at 1000rpm for 5 min. Remove the supernatant and resuspend the cells in 10 mL of FACS buffer.
4) Count the cells using trypan blue and hemacytometer.

#### Day-14: (II) Primary BMSC Cell Culture

1) Warm up complete media to 37°C in a water bath.
2) Isolate primary bone marrow cells and count the cells.
3) Centrifuge and resuspend the cells in complete media to obtain a cell concentration of 2 × 10^6^ cells/ml.
4) Seed 3 ml of cell solution in each well of a tissue culture treated 6-well plates to obtain a seeding concentration of 6 × 10^6^cells/well or 6.25 × 10^5^cells/cm^2^.
5) Grow cells for four days untouched in a cell incubator at 37°C, 5% CO_2_, and 95% humidity to let the BMSCs adhere.
6) On day 4, replace media with warm complete media to remove non-adhered cells, dead cells, and debris.
7) Expand BMSCs for another 7–10 days before using use. Replace media every 2–3 days.

#### Day-1: Reagent Preparation and Emulate Chip S-1 Chip Activation

1) Prepare chip activation and extracellular matrix solution according to the manufacturer’s instruction (Basic Organ-Chip Culture Protocol, Emulate EP177 v1.0, April 30, 2019, https://emulatebio.com/wp-content/uploads/2021/06/EP177_v1.0_Basic_Organ-Chip_Culture_Protocol.pdf).
2) Once activated, each cell compartment is added in a sequential manner according to the workflow timeline in Figure 1B.

#### Day 0: (I) BMSC Cell Seeding and Osteoblastic Cell Differentiation

1) Trypsinize BMSC cells in T-75 flasks using 5 mL of 0.25% Trypsin-EDTA for 3 min in a cell culture incubator.
2) Neutralize the trypsin by adding 20 mL of gas equilibrated osteoblastic differentiation media. Gently pipette the solution to release the cells and centrifuge the cells at 500 g.
3) Repeat trypsinization if there are still adhered BMSC on the culture flask.
4) Resuspend the cells in 10 mL of the same media for cell counting. Centrifuge the cells and remove the supernatant.
5) Resuspend the BMSCs in an appropriate volume of media to obtain a density of 2 × 10^6^ cells/mL.
6) Prepare Chip S-1 by flushing both the apical and basal channels with 100 μL of gas equilibrated media.
7) Remove the content of the apical channel using an aspirator and immediately pipette 45 μL of the cell solution to obtain a seeding density of 6 × 10^4^ cells/chip.
8) Invert the chips and place them in a sterile cell culture dish (150 mm × 25 mm) to allow BMSC to adhere on the top surface of the apical channel. Add 1 mL of sterile water in a 15 mL conical tube cap in the culture dish to maintain humidity.
9) Leave the chips in the cell culture incubator for 2 h.
10) After 2 h, check on BMSC adherence on the top surface of the apical channel under a light microscope to ensure 70–90% confluent seeding. Reseed the chips with BMSC if the seeding density is low.
11) Remove unadhered cells by pipetting about 200 ul of media at the inlet of the apical channel while simultaneously aspirating the outflow at the outlet channel. Do not fully aspirate the content of the channels. Repeat washing with the basal channel.
12) Prepare Emulate Pod™ by placing 3 ml of gas equilibrated osteoblastic differentiation media into the inlet reservoir of the apical channel. Fill the apical outlet, basal inlet, and outlet reservoirs with 300 μL of media.
13) Assemble the chip with the Pod™ and connect the system to the Zoe™ Culture Module according to the manufacturer’s instructions (Basic Organ-Chip Culture Protocol, Emulate EP177 v1.0, April 30, 2019—refer to Day 2: Chips to Pods and Pods to Zoe section).
14) Set the flow settings to 30 μL/h in the apical channel and no flow in the basal channel.
15) Differentiate cells for 14 days while checking for air bubbles in the channels every 2–3 days. Clear any air bubbles with gas equilibrated media.
16) Add gas equilibrated media into the apical channel inlet reservoirs every 2–3 days and remove the content of the outlet reservoir. Replace 300 μL of media into the reservoir to avoid drying out.

#### Day 0: (II) BMSC Culture Preparation

Isolate and culture bone marrow cells from C57BL/6 J to obtain BMSC on Day 14 for cell encapsulation. Follow protocols for primary bone marrow cell isolation and BMSC culture.

#### Day 10: C166 Cell Line Culture

1) Warm up complete endothelial media to 37°C in a water bath.
2) Thaw a vial of frozen C166 appropriately according to the manufacturer’s protocol. (https://www.atcc.org/products/crl-2581).
3) Resuspend cells in 10 mL of complete endothelial media and seed the cells in a T-75 culture flask.
4) Incubate cells for 2–3 days until reaching 70–80% confluency before use.
5) Cells can be passaged at a 1:5 or 1:10 ratio and stored appropriately in liquid nitrogen before use. Use C166 at passage 1-5.

#### Day 14: (I) Endothelial Cell Seeding

1) Trypsinize C166 endothelial cells in T-75 flasks using 5 mL of 0.25% Trypsin-EDTA for 3 min in a cell culture incubator.
2) Neutralize the trypsin by adding 20 mL of gas equilibrated complete endothelial media. Gently pipette the solution to release the cells and centrifuge the cells at 1000 rpm or 500 g. Resuspend the cells in 10 mL of media and count the cells.
3) Seed 25 μL of 2 × 10^6^cells/mL C166 cell solution into the basal channel of the chips similarly according to the BMSC seeding protocol.
4) Invert the chips to allow cells to adhere onto the membrane surface of the basal channel (Figure 1A). Leave the cells to adhere in the incubator for two hours.

#### Day 14: (II) Hydrogel Seeding and Coculture

1) Isolate bone marrow cells from 1 C57BL/6 J mouse according to the primary bone marrow cell isolation protocol. Count the cells and resuspend them in gas equilibrated supplemented DMEM media.
2) Trypsinize and collect the BMSC grown for 14 days. Count the cells and resuspend them in gas equilibrated supplemented DMEM media.
3) Thaw fibrin hydrogel components. Calculate the volume of each component needed for the number of chips to be seeded. Each chip will obtain 45 μL of fibrin gel solution containing whole bone marrow cells and BMSC in the apical channel. The final gel solution will have 5 mg/mL of fibrinogen, 25 μg/mL aprotinin, 0.2 mg/mL collagen type 1, and 0.5 U/mL thrombin.
4) To prevent premature crosslinking, prepare the gel solution in two separate mixes. Combine fibrinogen and aprotinin in tube A and collagen type 1 and thrombin in tube B.
5) Combine 6 × 10^4^ BMSC and 6 × 10^4^ bone marrow cells per chip and resuspend in the amount of media required to bring up the total volume to the desired final volume. Mix the cell solution with tube B.
6) For each chip, separately aliquot the desired volume tube A and tube B containing media with cells and immediately seed into the apical channel of Chip S-1. Check for bubbles. Reseed the apical channel in the presence of air bubbles.
7) Leave the chips in the cell incubator for 30 min to 1 h in a humidified cell culture dish for the gel to fully polymerize.
8) Remove the contents of the inlet and outlet reservoirs of the Pod™ and fill up 3 mL of gas equilibrated supplemented endothelial media in the basal channel inlet reservoirs. Add about 300 μL of media to the remaining reservoirs.
9) After the gel is polymerized, connect Chip S-1 to Pod™ and Zoë™. Setup flow at 30 μL/h in the basal channel where the endothelial cell monolayer is present.
10) Culture chip for another 7 days. Check for bubbles and replace the media every 2–3 days.

#### Day 21: Harvesting Cells

1) Prepare chip digestion solution.
2) Disconnect Chip S-1 from the Zoë™ and plug the outlet ports of the apical and basal channels with a 200 μL pipette tip.
3) Add 100 μL digestion solution to the apical channel and 50 μL digestion solution to the basal channel. Leave pipette tips in the inlet port.
4) Incubate the chips for 1 h in the incubator.
5) After 1 h, collect the contents of the apical and basal channel into a microcentrifuge tube.
6) Trypsinize the channels with 0.25% Trypsin-EDTA for 5 min at 37°C to collect the remaining cells on the membrane surfaces. Check for detachment under a light microscope.
7) Collect trypsin digest into the same collection tubes and add 1 mL of media to neutralize the trypsin.
8) Centrifuge the cell suspension to remove the digest media.
9) Resuspend cells in 1 mL of media and count the cells.
10) The cells are now ready for appropriate analysis steps.

### Flow Cytometry

1) C57BL/6 J marrow cell control. Isolate cells from each mouse using the previous cell isolation protocol. Cells can be stored in liquid nitrogen.
2) Resuspend the cells in a 5 mL 1 × RBC lysis buffer for 5 min at room temperature.
3) Wash with 5 mL FACS buffer. Centrifuge the cells and decant the supernatant.
4) Resuspend the cells in FACS buffer and set aside 1 × 10^6^ of isolated C57BL/6 J cells for DAPI and unstained compensation controls.
5) Prepare the primary lineage staining solution with the appropriate antibodies (Supplementary Table S1). Prepare the desired volume of master mix for 1 μL of each antibody solution per sample.
6) Centrifuge the harvested cells and controls in FACS tubes. Remove the supernatant.
7) Add 100 μL of lineage-biotin staining solution to each sample and leave the cells at 4°C for 25 min. In the meantime, prepare fluorophore-conjugated antibodies staining solution Supplementary Table S1.
8) Wash with staining solution by topping off the FACS tube with FACS buffer. Centrifuge the cells and decant the supernatant.
9) Repeat the staining process with fluorophore-conjugate antibodies solution. Leave the cells at 4°C for 25 min.
10) In the meantime, prepare flow cytometry compensation control for each fluorophore by combining 1 μL of conjugated antibody, 2 drops of UltraComp beads, and 200 μL of FACS buffer in a FACS tube. For lineage compensation, combine all four biotin-labelled lineage antibodies with fluorescently labelled streptavidin antibodies. For DAPI staining control, resuspend prepared C57BL6J cells with 200 μL of DAPI solution. Prepare unstained cells in 200 μL FACS buffer.
11) After staining is completed, wash the staining solution appropriately. Resuspend the cells in 200 μL of DAPI solution.
12) The cells are ready for flow cytometry analysis using the appropriate cell analyzer system. For the abovementioned experiments, cells were analyzed using BD LSRFortessa™ and BD FACS diva software. Gating analysis was performed using FlowJo v10.7. LSK sorting and *Ex-vivo* culture in BMME-on-chip

### *Ex Vivo* LSK Culture and Competitive Transplant

#### Day 0—Day 14(I): BMME-On-Chip Preparation

Generate chips with osteoblastic layer and endothelium layer according to BMME-on-chip preparation protocol: Day 14 to Day 14 (I).

#### Day 14: (II) Donor LSK Cells Sorting

1) Euthanize 4 C57BL/6 J CD45.2+ mice and isolate bone marrow cells appropriately.
2) Lyse RBCs as previously described for flow cytometric analysis.
3) Count the cells and prepare the desired volume of staining solutions (Supplementary Table S2). Set aside 1 × 10^6^ cells each for non-magnetically depleted control, unstained cells, and DAPI.
4) Stain the cells with magnetically-labelled lineage antibodies at 1:100 (Supplementary Table S2) for 25 min at 4°C.
5) Wash the cells with 2 mL of FACS buffer.
6) Resuspend in streptavidin-conjugated magnetic particles using volume corresponding to 50 μL per 1 × 10^7^ cells. Incubate the cells for 20 min at 4°C.
7) Add FACS buffer to the top of the volume of the tubes to 1 mL. Magnetically deplete the cells by placing the samples on a magnet for 4 min. Remove lineage negative supernatant into intermediate flow tube.
8) Resuspend magnetically labelled cells with 1 mL of FACS buffer again and repeat depletion.
9) Perform depletion one more time and transfer the supernatant to the final tubes.
10) Stain the depleted cells with fluorophore-conjugated antibodies for 25 min at 4°C (Supplementary Table S2). Prepare the compensation controls.
11) After staining, add 250 μL of FACS buffer to each sample tube. The samples are ready for flow sorting. Sort for HSPC (Lin-Sca1+cKit+). For the experiments above, cells were sorted using a FACSAria cell sorter (BD Biosciences).

#### Day 14: (III) LSK and BMSC Gel Seeding

1) Prepare gel solution as described in the BMME-on-chip preparation: Day 14(II).
2) Seed LSK cells at 3 × 10^4^cells and BMSC 6 × 10^4^ in fibrin hydrogel appropriately in the apical channel of prepared Chip S-1.
3) Culture cells under a continuous flow condition at 30 μL/h with supplemented DMEM media in the basal channel for another 14 days. Check for air bubbles and replace the media every 2–3 days.

#### Day 29: Primary Irradiation of Recipient Mice

1) Irradiate 10 B6 CD45.1+ mice with 5Gy of irradiation. Five mice will receive bone marrow cells from CD45.2+ mouse donors, and five mice will receive bone marrow cells from CD45.2+ chip cells (Figures 3B and C).

#### Day 28: (I) Harvesting Cells

1) Harvest the content of each cell according to the described protocol, Day 21: harvesting cells. The cell is ready for competitive transplant assay into B6 CD45.1+ recipients.
2) Isolate bone marrow cells from 1 B6 CD45.1+ mouse. These cells will be used as the competitor.
3) Isolate bone marrow cells from 1 C57BL/6 J mouse. These cells will be used as the control bone marrow donor.

#### Day 28: (II) Competitive Transplant

1) Prepare cell solution for competitive transplant with the following setup:

Chip Group: Each recipient will receive the total contents of 1 chip (CD45.2+ donor) and 1 × 10^5^ CD45.1+ competitor cells.

Control Group: Each recipient will receive a number of C57BL/6 J bone marrow cells (CD45.2+ donor) equivalent to the average number of cells obtained from the chips and 1 × 10^5^ CD45.1+ competitor cells.

Each mouse will receive a cell solution of 100 μL in FACS buffer.

2) Perform secondary irradiation of the ten recipient mice with 5Gy irradiation. Perform competitive transplant *via* tail-vein injection.
3) Collect 2–3 drops (∼200 μL) of peripheral blood at 4, 8, 12, 16, and 28-weeks post-transplant using submandibular bleeding technique. Analyze CD45.2+ donor engraftment using flow cytometry to detect CD3e+ T-lymphocytes, B220+ B-lymphocytes, and CD11b+ myeloid cells that are differentiated from CD45.1+ competitor cells (Supplementary Table S3).
4) Isolate bone marrow at 28-weeks post-transplantation (or earlier at 16-weeks) to analyze for CD45.2+ donor engraftment in HSPC population using flow cytometry (Supplementary Table S4).

### Alkaline Phosphatase and Von Kossa Staining

1) To analyze mineralization for osteoblastic differentiation on chips, fix chips by adding 10% neutral formalin buffer into the inlet of the apical (50 μL) and basal channels (20 μL). Fix cells at room temperature for 30 min.
2) Wash the cells with 1x distilled water for 5 min. Repeat 2–3 times.
3) Add prepared alkaline phosphatase staining solution into the apical and basal channel of each chip. Incubate for 45 min at room temperature.
4) Rinse in distilled water 3–4 times and leave in distilled water for 1 h.
5) Stain with 2.5% silver nitrate solution of 30 min.
6) Aspirate the solution and rinse with distilled water 3 times.
7) Image cells under a microscope or store them at 4°C. For the abovementioned experiments, images were taken using Biotek Cytation 5 Cell Imagine Multimode Reader at 10 × magnification with color brightfield. Mosaic imaging and stitching were automated using Biotek Gen5 imaging and analysis software.

### Image Analysis Using ImageJ

Image analysis of the alkaline phosphatase and Von Kossa stained chips was performed using ImageJ software, as described in Supplementary Figure S1 **.** Immunofluorescent staining and imaging were performed as described in Supplementary Figure S2. Image analysis of ZO-1 and DAPI staining was performed in ImageJ, as described in Supplementary Figure S3.

### Statistical Analysis

All statistical analyses were performed using GraphPad Prism 9 statistical analysis software. For all quantitative results, groups are reported as mean ± standard deviation (SD). For comparisons between two groups, statistical analysis was performed using student’s unpaired two-tailed *t-*test for normally distributed. Otherwise, unpaired and non-parametric multiple *t-*tests were performed with Mann–Whitney post-test. For comparisons between two groups across timepoints (Figure 5), statistical analysis was performed using restricted maximum likelihood (REML).

## Anticipated Results

We anticipate that the modular nature of this BMME-on-chip will allow for rigorous analysis of individual cellular components of the bone marrow microenvironment in a model that more closely recapitulates the *in situ* HSC niche. The modular nature of this model and the ability to use different cell populations from unique sources and genetic backgrounds is a powerful tool. We anticipate that this system will be used to clarify the roles of individual cell populations of the BMME during hematopoietic homeostasis, as well as during aging, and in pathological states such as leukemia and multiple bone marrow failure syndromes.

## Limitations

The described murine BMME-on-chip provides validation that an *in vitro* system is capable of maintaining multi-potent and self-renewing HSCs for at least 14 days. Competitive transplantation of HSCs into an immune competent recipient is the gold standard for quantifying HSC function. As this analysis is not possible with the use of human cells, this represents a strength of the murine system in validation prior to the use of a fully human BMME-on-chip. However, it also represents a limitation as mouse models do not always recapitulate the human environment. Previously published BMME-on-chip devices that use human tissue have demonstrated the ability to maintain phenotypic HSPCs (Chou et al., 2020). Therefore, it is likely that the functional ability to self-renew and repopulate the hematopoietic system of a myeloablated recipient that we report here in the murine model is translatable to a human environment.

While our murine BMME-on-chip includes multiple key components of the bone marrow HSC niche, it is not fully representative of the physiologic bone marrow. For example, osteoclastic bone resorption is an ongoing process *in vivo* and is a component not included in our system. In addition, osteocytes embedded in bone are the most abundant bone cell *in vivo* and are not present in our BMME-on-chip (Frisch, 2019). The relative importance of these cells and processes for the regulation of HSCs are not well-established, and thus the implication of their absence in our system is not known (Soto et al., 2021). However, the ability of our BMME-on-chip to maintain a fully functional HSC population suggests that they are not required for HSC maintenance.

## Conclusion

In leukemia such as acute myeloid leukemia (AML) and chronic myeloid leukemia (CML), leukemic cells can interact with the surrounding stroma to create a favorable environment for leukemia stem cell LSC maintenance and expansion of leukemic cells (Frisch et al., 2012; Shafat et al., 2017; Behrmann et al., 2018; Staversky et al., 2018; Xu et al., 2021). The BMME is made up of cellular and noncellular components such as BMSC, endothelial cells, osteoblasts, osteoclasts, fibroblasts, adipocytes, macrophages, and extracellular matrix that provide crucial signals for the homeostatic regulation of hematopoietic systems. Leukemia has been shown to interact with the BM niche by modulating endothelial activation, establishing crosstalk with fibroblasts, and inhibiting osteoblasts (Ryningen et al., 2005; Frisch et al., 2012; Pezeshkian et al., 2013; Krevvata et al., 2014; Shafat et al., 2017; Staversky et al., 2018). In particular, our group and others have shown that leukemic cell interaction with osteoblasts is an important component in sustaining leukemic burden. However, the interactions of the HSC niche with bone marrow pathologies are poorly understood (Soto et al., 2021). AML is the most common form of leukemia that arises in the bone marrow (Soto et al., 2021). The standard therapy for AML of combined daunorubicin and cytarabine administration, termed 7 + 3 induction therapy, which was first employed in the 1970s, is still the predominant upfront therapy for most AML patients and gives rise to poor outcomes with an overall 5-years survival rate of <30% (Roussel et al., 2020; Kantarjian et al., 2021). Therefore, a better understanding of the role of the BMME in AML is critical for the improvement in therapy and patient outcomes.

The use of a BMME-on-chip that recapitulates many of the cellular interactions that occur in the BMME *in vivo* will allow for rigorous analysis of the BMME during hematologic malignancies. A murine environment, like the one in the BMME-on-chip described herein, will validate that phenotypes identified *in vivo* murine leukemic models can be recreated in an *in vitro* environment, and suggest that the *in vitro* environment of a similar human BMME-on-chip will recreate human bone marrow phenotypes that are not possible to directly observe *in vivo*. Therefore, this BMME-on-chip microphysiological platform will serve as a critical pre-clinical model for the identification of novel therapeutic targets.

## Data Availability

The raw data supporting the conclusion of this article will be made available by the authors, without undue reservation.
