# Supplementary material for: Bone Marrow Microenvironment-On-Chip for Culture of Functional Hematopoietic Stem Cells
Source: Front Bioeng Biotechnol. 2022 Jun 20;10:855777. doi: 10.3389/fbioe.2022.855777 (PMC9252162; doi:10.3389/fbioe.2022.855777)

Supplemental Table 1: HSPC flow cytometry panels

HSPC antibodies					
**Staining solution I: Lineage-Biotin antibodies**					
Antibodies	Label	Reactivity	Clone	Manufacturer	Cat#
Gr1	Biotin	anti-mouse	RB6-8C5	eBioscience	13-5931-82
B220 (CD45R)	Biotin	anti-mouse/human	RA3-6B2	eBioscience	50-115-52
CD3e	Biotin	anti-mouse/human	145-2C11	eBioscience	13-0031-82
Ter119	Biotin	anti-mouse	TER-119	eBioscience	13-5921-82
**Staining solution II: Fluorophore-conjugated antibodies**					
Antibodies	Label	Reactivity	Clone	Manufacturer	Cat#
Sca1	PerCP Cy5.5	anti-mouse	D7	Biolegend	108123
cKit/CD117	PeCy5	anti-mouse/human	2B8	eBioscience	15-1171-82
CD48	APC Cy7	anti-mouse	HM48-1	eBioscience	17-0481-82
CD150	BV785	anti-mouse	TC15-12F12-2	Biolegend	115937
Flt3 (CD135)	PE	anti-mouse	A2F10	eBioscience	12-135182
Streptavidin	BV650	anti-biotin		Biolegend	405231
**Staining solution III**					
0.1µg/ml DAPI solution in 1X PBS, Invitrogen Cat# D21490					

Supplemental Table 2: LSK Sorting Panel

LSK antibodies					
**Staining solution I: Lineage-Biotin antibodies**					
Antibodies	Label	Reactivity	Clone	Manufacturer	Cat#
Gr1	Biotin	anti-mouse	RB6-8C5	eBioscience	13-5931-82
B220 (CD45R)	Biotin	anti-mouse/human	RA3-6B2	eBioscience	50-115-52
CD3e	Biotin	anti-mouse/human	145-2C11	eBioscience	13-0031-82
Ter119	Biotin	anti-mouse	TER-119	eBioscience	13-5921-82
**Staining solution II: Streptavidin magnetic particles**					
Streptavidin-iMAG, BD Biosciences, Cat# 557812					
**Staining solution III: Fluorophore-conjugated antibodies**					
Antibodies	Label	Reactivity	Clone	Manufacturer	Cat#
Sca1	PerCP Cy5.5	anti-mouse	D7	Biolegend	108123
cKit/CD117	PeCy5	anti-mouse/human	2B8	eBioscience	15-1171-82
Streptavidin	BV650	anti-biotin		Biolegend	405231
**Staining solution IV**					
0.1μg/ml DAPI solution in 1X PBS, Invitrogen Cat# D21490					

Supplemental Table 3: Competitive Transplant Blood Cells Flow Panel

Blood cells antibodies					
**Staining solution I: Fluorophore-conjugated antibodies**					
**Antibodies**	**Label**	**Reactivity**	**Clone**	**Manufacturer**	**Cat#**
CD3e	PerCP-Cy5.5	anti-mouse	145-2C11	BD Biosciences	BDB561108
CD11b	APC Cy7	anti-mouse	M1/70	BD Biosciences	BDB561039
B220/CD45R	APC	anti-mouse	RA3-6B2	BD Biosciences	BDB561880
CD45.1	PE	anti-mouse	A20	BD Biosciences	BDB561872
CD45.2	FITC	anti-mouse	104	BD Biosciences	BDB561874
**Staining solution II**					
0.1µg/ml DAPI solution in 1X PBS, Invitrogen Cat# D21490					

Supplemental Table 4: Competitive Transplant Bone Marrow HSPC Panel

Bone marrow HSPC antibodies					
**Staining solution I: Lineage-Biotin antibodies**					
**Antibodies**	**Label**	**Reactivity**	**Clone**	**Manufacturer**	**Cat#**
Gr1	Biotin	anti-mouse	RB6-8C5	eBioscience	13-5931-82
B220 (CD45R)	Biotin	anti-mouse/human	RA3-6B2	eBioscience	50-115-52
CD3e	Biotin	anti-mouse/human	145-2C11	eBioscience	13-0031-82
Ter119	Biotin	anti-mouse	TER-119	eBioscience	13-5921-82
**Staining solution II: Fluorophore-conjugated antibodies**					
**Antibodies**	**Label**	**Reactivity**	**Clone**	**Manufacturer**	**Cat#**
Sca1	PerCP Cy5.5	anti-mouse	D7	Biolegend	108123
cKit/CD117	PeCy5	anti-mouse/human	2B8	eBioscience	15-1171-82
CD48	APC Cy7	anti-mouse	HM48-1	eBioscience	17-0481-82
CD150	BV785	anti-mouse	TC15-12F12-2	Biolegend	115937
Flt3 (CD135)	PE	anti-mouse	A2F10	eBioscience	12-135182
Streptavidin	BV650	anti-biotin		Biolegend	405231
CD45.1	APC	anti-mouse	A20	BD Bioscience	50-112-3055
CD45.2	FITC	anti-mouse	104	BD Bioscience	BDB561874
**Staining solution III**					
0.1µg/ml DAPI solution in 1X PBS, Invitrogen Cat# D21490					

Supplemental Figure 1: Imaging analysis for Alkaline Phosphatase and VonKossa Staining

Image processing

(A) Color

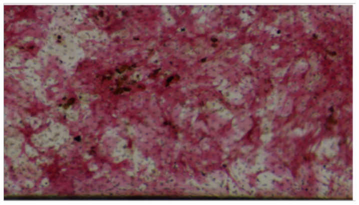

1. Import stained images from Cytation 5 Imager into ImageJ.
2. Crop images to only show area containing cells. Note: image (A) only displays a small portion of the cropped area.

(B) 16-bit and B&C

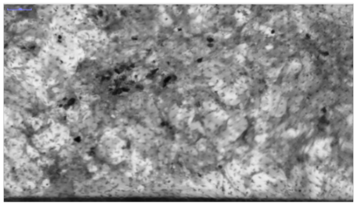

3. Convert images to 16-bit.
4. Adjust Brightness and Contrast using the auto setting and apply to all images. Note: (B) Images for this experiment were set to B&C values of Min 11 and Max 137.

(C) Threshold

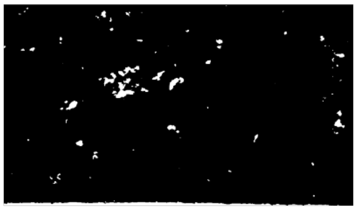

5. Adjust threshold using Huang method Note: (C) All images were set to Min 64 and Max 255.

(D) Binary + dilate

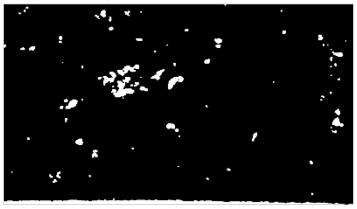

6. Set images to Binary and apply Dilate.

(E) Watershed

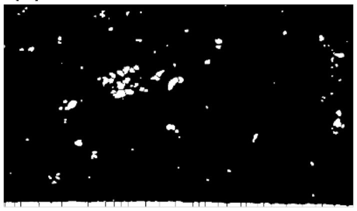

7. Apply Watershedding.

(F) Analyze particle

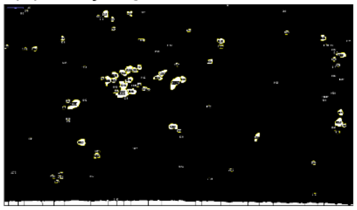

8. Use the Analyze Particle tool to calculate number and size of calcium nodules. Choose exclude boundary option.
9. Import data into GraphPad Prism for further analysis. Note: (F) Values below 40pixel^2 were excluded to remove artifacts from membrane pores.

Supplemental Figure 2: ZO-1 Staining protocol and 2D monolayer control staining

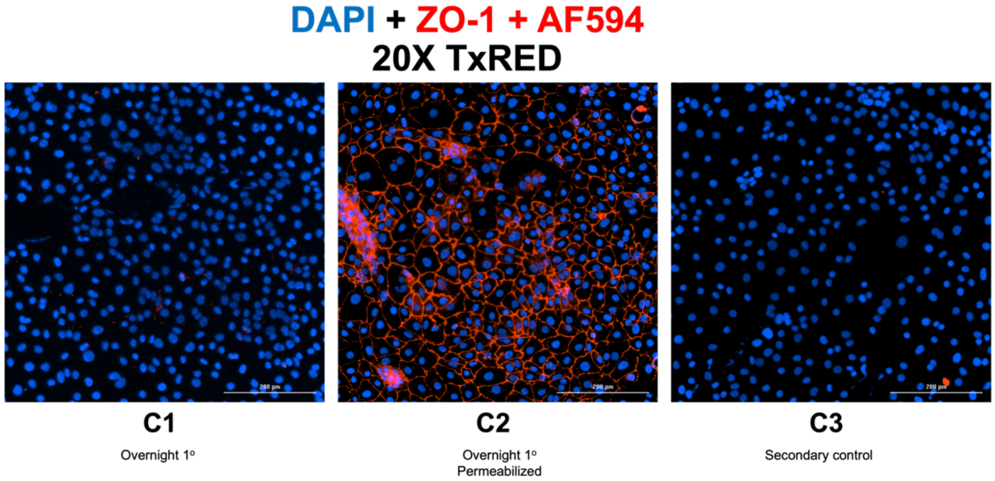

Staining Protocol

1. Fix endothelial cells in chips with 10% Normal Buffered Formalin for 20 minutes at room temperature.
2. Aspirate content of chips and wash by submerging in 1X PBS in a petri dish for 5 minutes. Flow PBS in the channels 1-2 times using an aspirator. Repeat 3x
3. Store chips in 1% BSA (fraction V) in 1X PBS (1% PBSA) at 4°C until staining.
4. Permeabilize cells with 0.1% Triton X-100 (XXXXX) in 1x PBS for 15 minutes at room temperature.
5. Wash chips with PBS for 5 minutes. Repeat 3x.
6. Block with 3% BSA in 1X PBS for 1 hour at room temperature.
7. Stain with ZO-1 antibody in 1% PBSA overnight at 4°C.

    Mouse Anti-ZO-1 Monoclonal Antibody
    (ZO1-1A12, Invitrogen Cat# 33-9100)
    Dilution 1:100
8. Wash chips 3x with 1X PBS the following day.
9. Stain with secondary antibody for 1 hour at room temperature protected from light.

    Goat Anti-Mouse IgG AlexaFluor 594
    (Invitrogen Cat# A-11005)
    Dilution 1:1000
10. Wash chips 3x with 1X PBS.
11. Stain with DAPI at 15μg/ml in 1X PBS for 5 minutes at room temperature protected from light.
12. Wash chips 3x with 1X PBS.
13. Aspirate PBS completely from chips.
14. Add 90% glycerol in 0.1M Tris Buffer for fluorescent imaging.

Note: Images of ZO-1 and DAPI staining were performed using Biotek Cytation 5 Cell Imaging Multi-Mode Reader with DAPI and Texas Red led and filter sets.

Supplemental Figure 3: Image processing steps for ZO-1 and DAPI staining using FIJI.

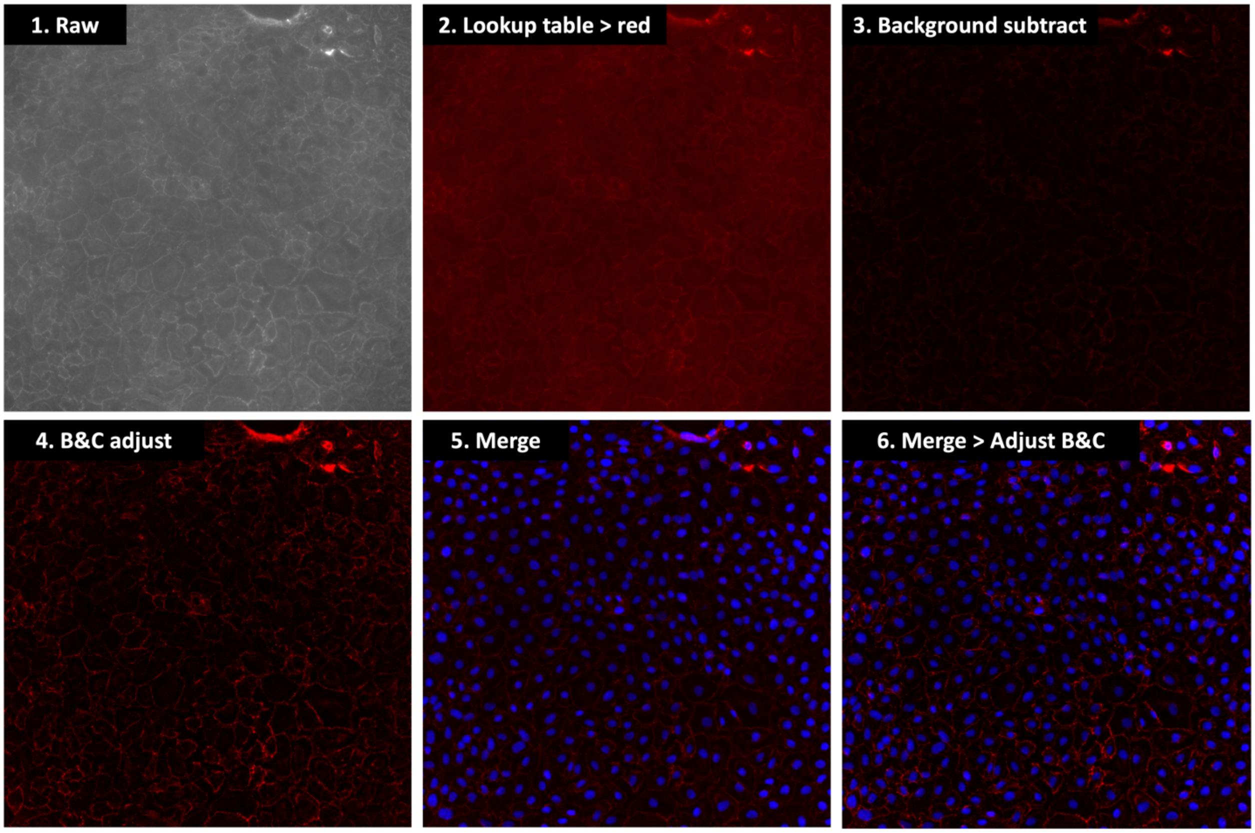

Supplemental Figure 4: Flow cytometry gating for myeloid and lymphoid cells in donor CD45.2+ cells in peripheral blood.

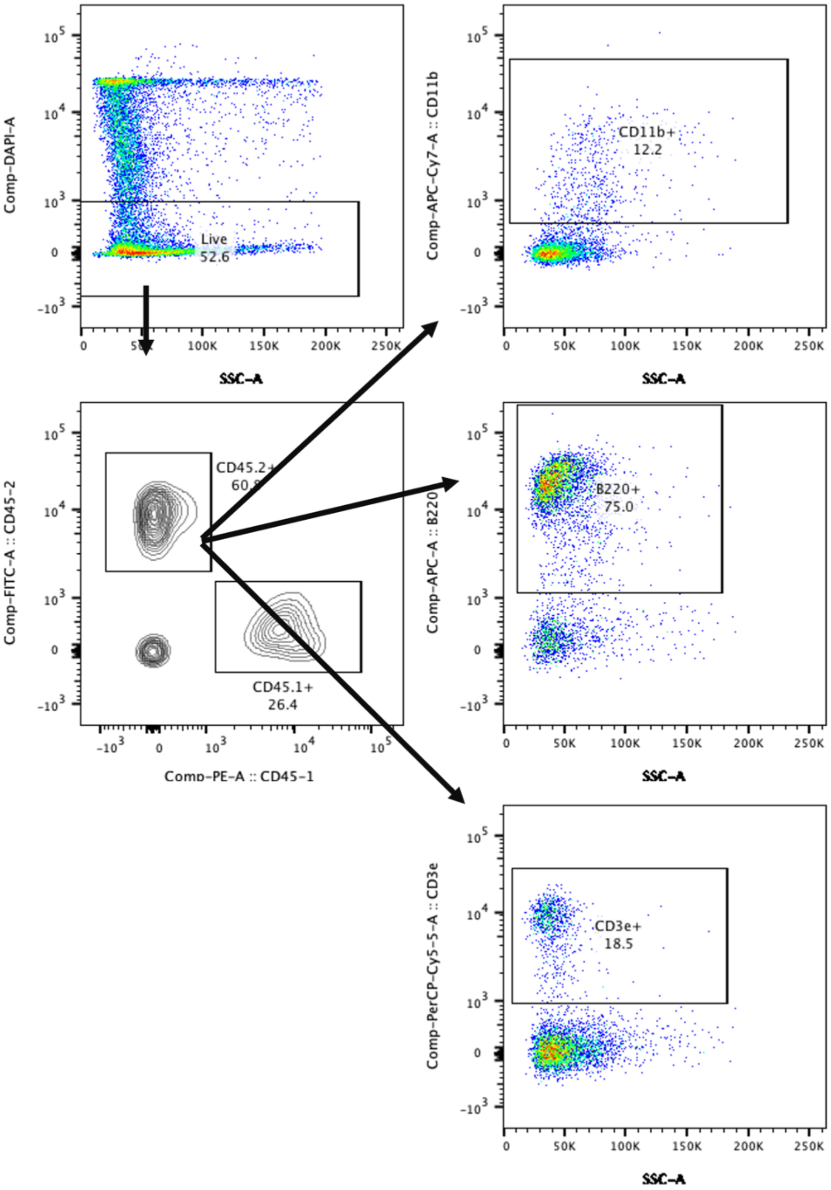

Supplement: Supplementary file 1 [file DataSheet1.PDF]
